# Supplementary material for: A qualitative study of the first batch of medical assistance team’s first-hand experience in supporting the nursing homes in Wuhan against COVID-19
Source: PLoS One. 2021 Apr 15;16(4):e0249656. doi: 10.1371/journal.pone.0249656 (PMC8049312; doi:10.1371/journal.pone.0249656)
Supplement: S1 Appendix — (DOCX) [file pone.0249656.s001.docx]

# S1 Appendix.

# The date of “A qualitative study of the first batch of medical assistance team's first-hand experience in supporting the nursing homes in Wuhan against COVID-19”

# Interview outline

**Table 1 Interview questions for the first-line medical support specialists**

| **Interview questions** |
| --- |
| - Could you please talk about what you have done in supporting the nursing homes in Wuhan to fight the COVID-2019? - In your process of understanding and on-the-spot guidance to the nursing homes in Wuhan, what do you think are the difficulties encountered by the nursing homes? How were resolved? |
| - Do you feel pressured during the process of guiding the fight against the COVID in the nursing homes in Wuhan? If so, what are your pressures? How did you solve them? |
| - What are the most impressive things in the process of supporting the nursing homes in Wuhan? Could you share with examples? |
| - In this anti-epidemic process, what kind of services do you think we need to provide for the elderly? Can you give an example? |
| - What do you think are the aspects of the nursing homes in Wuhan that have done a good job in the fight against the epidemic? What needs to be improved? |
| - How does this incident inspire the nursing homes to respond to such public health emergencies in the future? |

# Participants

We conducted online video interviews with seven medical staff supporting the nursing home in Wuhan from March 25 to 31, 2020. Characteristics of the seven participants were shown in table 2. Among them, two were professional fields of public health, two were managers of western medicine physicians, two were front-line clinical nurses and one was clinical doctor of traditional Chinese medicine. The average age is 47 years (26-60). There were three males (42%), four females (58%). The average working days were 30 days, and the average working years were 20 years.

**Table 2 General information of research objects**

| **Code** | **Age** | **Sex** | **Education level** | **Field of work** | **Professional level** | **Professional years** | **Support Wuhan time** | **Support time in Wuhan** |
| --- | --- | --- | --- | --- | --- | --- | --- | --- |
| **P1** | 48 | F | Bachelor degree | Administrator (Public health management) | Intermediate title | 29 years | Feb 22  (2020) | 30 days |
| **P2** | 60 | M | Doctor degree | Administrator (Western medicine physicians) | Senior title | 37 years | Feb 20  (2020) | 33 days |
| **P3** | 51 | F | Bachelor degree | Administrator (Public health management) | Technical master | 29 years | Feb 27  (2020) | 27 days |
| **P4** | 47 | M | Bachelor degree | Administrator（Western medicine physicians） | Intermediate title | 23 years | Feb 23  (2020) | 31 days |
| **P5** | 29 | M | Bachelor degree | Doctor of traditional Chinese Medicine | Junior title | 5 years | Feb 23  (2020) | 33 days |
| **P6** | 31 | F | Bachelor degree | Nurse | Senior title | 10 years | Feb 22  (2020) | 30 days |
| **P7** | 26 | F | Bachelor degree | Nurse | Junior title | 6 years | Feb 22  (2020) | 30 days |

F, Female; M, Male.

Note: The coding is in the order of interview and has nothing to do with other information.

Based on our literature review and interviews, we extracted five generic categories and 19 sub categories about the experience of fighting COVID -19 in nursing homes (Table 3), as follows:

**Table 3 The category of content analysis.**

| **Main theme** | **Sub-theme** |
| --- | --- |
| Difficulties Faced by the Nursing Homes | Nursing assistants lack of knowledge |
|  | Lack of equipment |
|  | Lack of experience |
|  | Shortage of staff |
| Psychological Experience | Pressure from many sources |
|  | Team-cooperation |
|  | Challenge |
| Service for the Older People | Providing epidemic prevention knowledge |
|  | Traditional Chinese medicine health care |
|  | Mental consolation |
|  | Basic daily care |
| Prevention and Management Strategies | Establish contingency plans |
|  | Health assessment |
|  | Targeted training |
|  | Single isolation |
| Strategies for Public Health Emergencies | Physical space design |
|  | Routine training |
|  | Routine training |

**Main theme 1: Difficulties Faced by the Nursing Homes**

**Sub-theme 1: Nursing assistants lack of knowledge**

The staff of the organization had weak awareness of epidemic prevention, and the work of epidemic prevention is not standardized:

*"When we arrived at the support organization, we found that the organization did not carry out strict in and out management, and there were three career who could go home, because there were elderly people in need of care" (participant 1).*

The protection of the organization's staff is unqualified, the wearing and taking off protective equipment such as isolation clothing, masks and goggles, do not meet the professional standards:

*"The organization should strictly implement the three-level prevention and control of infectious diseases, which means that the clothing is double-layer, that meaning, isolation clothing, protective clothing, masks and goggles, gloves and shoe covers are two pairs. A suit of protective clothing could be used for three or four days, and some people washed the protective clothing with water" (Participant 1).*

Sub-theme 2: Lack of equipment

Isolation clothing, masks, goggles and other isolation equipment specifications did not meet professional protection standards in a limited number:

*“At their worst, the paramedics were also infected and moved away, there was a severe shortage of staff and supplies, and they might just have a mask that they didn’t even wear gloves, and that’s really how they came into contact with the infected elderly” (participant 4).*

These nursing homes all lacked three areas, two channels and isolation observation rooms:

*“There were no three areas, two channels and isolation observation room in the nursing home, once confirmed, they would be sent to the designated hospital for treatment” (participant 5).*

Sub-theme 3: Lack of experience

The outbreak of COVID-19 is sudden. Everyone is slowly exploring its prevention and control, and there is no authoritative and feasible standard:

*"The prevention and control of the epidemic in the elderly care institutions had never been touched before, so we had no experience. All of us were going in teams on a temporary basis, there was not enough communication and tacit understanding between teams" (participant 6).*

Sub-theme 4: Shortage of staff

Most of the care staff in the nursing homes, affected by their own cultural level and professional fields, can’t provide timely and accurate standard care for the elderly and self-protection during the epidemic:

*“In the existing institutions, there were a lot of nursing assistants, who may not have many educational opportunities at the cultural level. In the process of implementing management standard of hospital infection control, they had not strong cognition of hospital sense, therefore, the implementation did not conform to the specifications” (participant3).*

## Main theme 2: Psychological Experience

Sub-theme 1: Pressure from many sources

During the anti-epidemic period, the main pressure mainly includes two groups, the elderly and the staff of the institution. Managers use the Chinese version of self rating anxiety scale [17] to evaluate the psychological status of the elderly and staff, and found that most of them suffer from varying degrees of anxiety.

*A manager said that : “During the period of isolation, we randomly selected 90 elderly people with clear cognition from the institution for evaluation, and found that 27 of them were at severe anxiety, 39 were at moderate anxiety, and the remaining 24 were at mild anxiety. Among the 47 staff members, 35 person were at moderate anxiety, 8 person were at mild anxiety, and only 4 person no anxiety” (Participant 1).*

The older people were worried about being infected because someone nearby was diagnosed and sent away.

*"There were some old people who could not fully understand our prevention and control requirements, theory they all understood, but once this measure was implemented on them, thy would be impatient, anxious, swear and cry, so some old people could only limited cooperation, some old people completely did not cooperate” (Participant 3).*

The pressure of staff mainly comes from the support institutions and their daily work. Long time high intensity and high load work, physical overdraft, tiredness and psychological burden:

*"In fact, our pressure was mainly two aspects, the first was the prevention and control of the epidemic situation in pension institutions, we had not been exposed before, so there was no great assurance. The second was that the epidemic prevention work was not like charging on the battlefield, in the battlefield you go forward, not afraid of death are no problem. If one of our players infected, it means all of us members of the team couldn’t do any work and to be sequestered” (Participant 1).*

Sub-theme 2: Team-cooperation

The support staff mainly includes four categories: doctors, nurses, nursing assistants for the aged and civil servants of the government.

*“After we arrive in Wuhan, if some policies or processes are difficult to be widely used, we need to use the superior departments. When the work is promoted, if there is any problem or contradiction, the government department will directly order and urge the following implementation” (Participant 6).*

It is also important to share experiences among different support teams.

*“because we had achieved remarkable results in the support process, the government asked us to share their experience with Wuhan Qiaokou District Welfare Institute and participated in the formulation of some rules and procedures of the Institute” (Participant 5).*

Sub-theme 3: Challenge

As medical professionals, combined with own field skills, scientific protest, in the process of anti-epidemic advice, give correct and effective guidance:

*“After we arrived at the nursing home, we found that the institution epidemic prevention and disinfection work was unqualified, I used to work in the department as a head of infection management, responsible for disinfection and isolation, familiar with infection management work, assessment of institution epidemic prevention work not in place, to help the institutions leaders” (participant 3).*

## Main theme 3: Service for the Older People

Sub-theme 1: Providing epidemic prevention knowledge

Most of the elderly in the institution are blind to related preventive knowledge, we combined the characteristics of the elderly to explain the knowledge of infectious diseases and protective measures:

*"After the training, the elderly improved their consciousness, and then the disinfection and isolation were standard. Our colleagues would pass some better news information about COVID-19 to their old people every day when they went to work, and then they would teach the elderly learn some knowledge about the prevention of virus and guide them to do well in self-protection" (Participant 7).*

Sub-theme 2: Traditional Chinese medicine health care

In the process of fighting the epidemic, traditional Chinese medicine has played a certain role in the treatment and prevention:

*"During the epidemic, it was forbidden for the elderly to go out for activities. We would teach the elderly health care points, let them massage themselves, teach them moxibustion methods, or help them with moxibustion when everyone's protection was qualified. Then there was the health preserving skill of traditional Chinese medicine, Baduanjin, which could help the elderly adjust their physical and mental state".*

Sub-theme 3: Mental consolation

To solve pressures faced by the elderly, it is important to take effective measures:

*“Considering that these elders had been isolated for more than 40 days, we also used many ways to give them some psychological comfort…...so we had created some conditions in this area. In addition, we download some graffiti from the Internet – Secret Garden, then guided the elderly to paint in their own room, and then made some handmade origami paintings” (Participant1).*

Sub-theme 4: Basic daily care

In simple basic nursing, the work of nursing assistants is taken over by professionals with relevant medical background:

*“Our staff in Wuhan and the previous work content was different, the main task was disinfection and sterilization, floor disinfection and sterilization. Floors, elevators, corridors, handrails, these disinfection work. The second one was to deliver meals to the elderly. After we picked up the three meals a day in the elevator, we would deliver them to the elderly’s room, then one was the old people’s drugs. For some old people with multiple chronic diseases, they need to dispense drugs” (Participant 1).*

## Main theme 4: Prevention and Management Strategies

Sub-theme 1: Establish contingency plans

The participants showed formulating service specifications, standard processes and relevant manuals, so as to make everyone clear their responsibilities and work rules was essential:

*"I think the most outstanding work done by our team, focused on the development of epidemic prevention standards and work processes, through the work norms, risk aversion, and were picturesque, such as the removal of the protective clothing this step all picture and text attached to the wall, the corridor paste a string of walk, you walked over, you could complete the removal of this protective clothing (see the Appendix)” (Participant 1).*

Sub-theme 2: Health assessment

Most of the elderly in institutions suffer from basic diseases such as chronic diseases, and may have many different symptoms. During the epidemic period, we should do a good job in supervision and comprehensive evaluation.

*"During the epidemic period, a grandmother with cerebral infarction always said that she was suffering from physical pain one night. At the time of shift handover, a nurse suddenly found out that it was wrong, and suggested that the doctor do a brain CT, which found that there was a thrombus in the cerebral blood vessels, and made corresponding treatment in time" (Participant 1).*

Sub-theme 3: Targeted training

The training targets are mainly divided into three categories, management, medical workers, and institutional logistics support (such as nursing assistant, doormen, cleaners, cooks, etc.):

*"We were basically put all the institutional processes on their walls, and they've got them all on the wall, and we had led them to talk and do it, and they were supposed to be able to learn to stick some of the more important points on the wall. All systems, all processes, that was, traditional protective processes, were all posted to them in a unified corridor, and they learn by themselves" (Participant 5).*

Training begins immediately after the team of experts has developed relevant processes and systems in the context of the organization:

*"In terms of personnel supervision, most of them were nursing assistants, because their overall knowledge of nursing assistants was weak and their knowledge of prevention and control was relatively lacking. So, we first did one thing, full staff training, including the relevant knowledge of COVID-19 and related knowledge of protection, spread the relevant knowledge" (Participant 4).*

Sub-theme 4: Single isolation

For confirmed cases immediately sent to designated hospitals for isolation treatment, their previous living environment is strictly sterilized and sterilized:

*"When I first found out that there was an old man who was diagnosed, all the old people were living in different layers at the first time. No old people lived in the same room. At the beginning, they were two old people living in the same room. Then they had a confirmed case at the first time and isolated all the old people like a single room" (Participant 7).*

Sub-theme 5: Restrict visitor

Strict entry and exit management system, entry and exit personnel, vehicles and materials must be strictly controlled:

*"For those who had to go in and out of the agency, vehicles carrying supplies were registered and temperature measured. The entry personnel block the face, by the special person used 500 mg/L chlorine disinfectant top-down Z shape to spray the whole body and the sole and carries on the hand disinfection to enter……" (Participant 2).*

## Main theme 5: Strategies for Public Health Emergencies

Sub-theme 1: Physical space design

The nursing home must be equipped with three areas and two channels when they are established. The distance between floors should not be too close:

*“So when I talk about the old facilities of the nursing home, I mean that, when they design pension institutions, they do not better involve human concepts” (Participant 3).*

Sub-theme 2: Personal allocation

The staff of the organization should be in a proper proportion. Medical staff and nurses should form a small team to serve the elderly together.

*“The organization should have medical staff, only nursing assistants and managers are not enough, they do not have enough professional knowledge to face public health events such as epidemic” (Participant 2).*

Sub-theme 3: Routine training

Regular training and emergency plan drill shall be conducted at ordinary times to clarify emergency measures, prevention and control process:

*"These materials for killing may need to be stored. Then there are even special personnel to train the use and operation of these things, and there are training and supervision, which may have been used as a normalized epidemic outbreak season, and then as a normalized killing" (Participant 5).*
